# Extracellular Matrix Modulates Angiogenesis in Physiological and Pathological Conditions

**DOI:** 10.1155/2014/756078

**Published:** 2014-05-18

**Authors:** Anna Neve, Francesco Paolo Cantatore, Nicola Maruotti, Addolorata Corrado, Domenico Ribatti

**Affiliations:** ^1^Rheumatology Clinic, Department of Medical and Surgical Sciences, University of Foggia, Ospedale “Col. D'Avanzo”, 71121 Foggia, Italy; ^2^Department of Basic Medical Sciences, Neurosciences and Sensory Organs, Section of Human Anatomy and Histology, University of Bari Medical School, 70124 Bari, Italy; ^3^National Cancer Institute “Giovanni Paolo”, 70124 Bari, Italy

## Abstract

Angiogenesis is a multistep process driven by a wide range of positive and negative regulatory factors. Extracellular matrix (ECM) plays a crucial role in the regulation of this process. The degradation of ECM, occurring in response to an angiogenic stimulus, leads to degradation or partial modification of matrix molecules, release of soluble factors, and exposure of cryptic sites with pro- and/or antiangiogenic activity. ECM molecules and fragments, resulting from proteolysis, can also act directly as inflammatory stimuli, and this can explain the exacerbated angiogenesis that drives and maintains several inflammatory diseases. In this review we have summarized some of the more recent literature data concerning the molecular control of ECM in angiogenesis in both physiological and pathological conditions.

## 1. Introduction

The extracellular matrix (ECM) is the noncellular component present within all tissues and organs, consisting of a variety of structural and signalling molecules secreted from differentiated mesenchymal cells including chondrocytes and fibroblasts and with biochemical, biomechanical, and structural properties critical for the development of organs.

**Figure 1:**
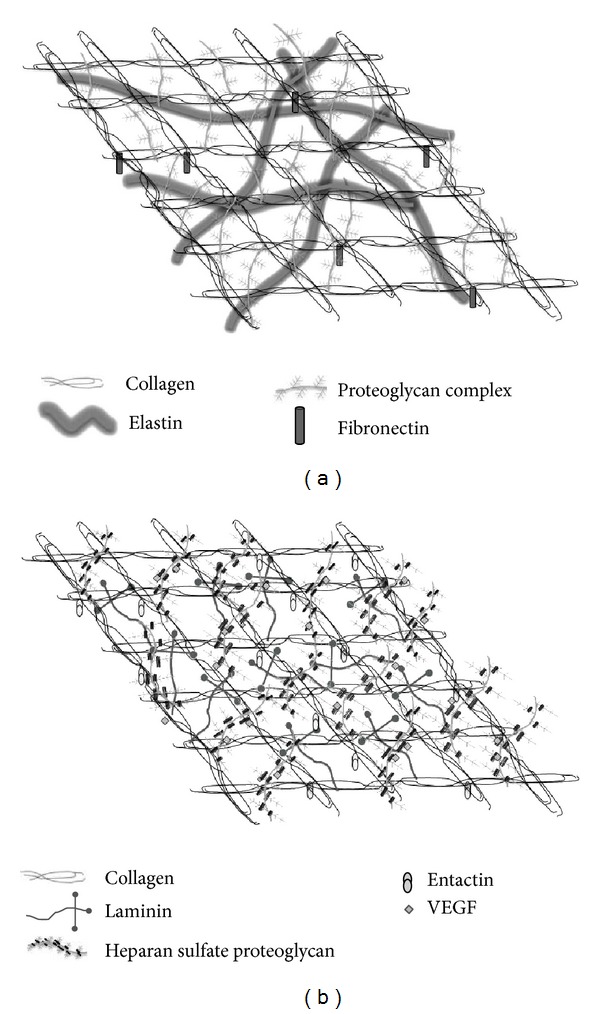
A schematic drawing of the extracellular matrix molecular organization. The interstitial matrix is mainly composed of collagen, fibronectin, elastin, and proteoglycans (a). The extracellular basement membrane mainly consists of collagen IV, laminin, entactin, and heparan sulfate proteoglycans which bind to VEGF (b).

The ECM provides mechanical adhesive support for the cellular constituents, directs their morphological organization, and influences physiological functions, by binding growth factors and interacting with cell-surface receptors. Two biochemically and morphologically differentiated entities have been identified: the interstitial matrix and the extracellular basement membranes (BMs). The first one is mainly composed of fibrillar and nonfibrillar collagens, elastic fibers, and glycosaminoglycan- (GAG-) containing noncollagenous glycoproteins (hyaluronan and proteoglycans) (Figure 1(a)). The BMs are highly specialized extracellular matrix sheets underlining epithelial or endothelial cells, consisting of collagen IV, laminins, entactin, and heparan sulfate proteoglycans (Figure 1(b)), which affect cell shape, gene expression, proliferation, migration, and apoptosis.

The ECM is a highly dynamic structure, undergoing continuous remodelling, which consists in the deposition, degradation, and modification of its components. An abnormal ECM dynamic leads to pathological processes including tissue fibrosis and cancer.

The three-dimensional (3D) and computational* in vitro* studies [1, 2] clearly demonstrate that besides its remodelling ECM controls and regulates physiological and pathological angiogenesis [3] at several levels by several ways.

**Figure 2:**
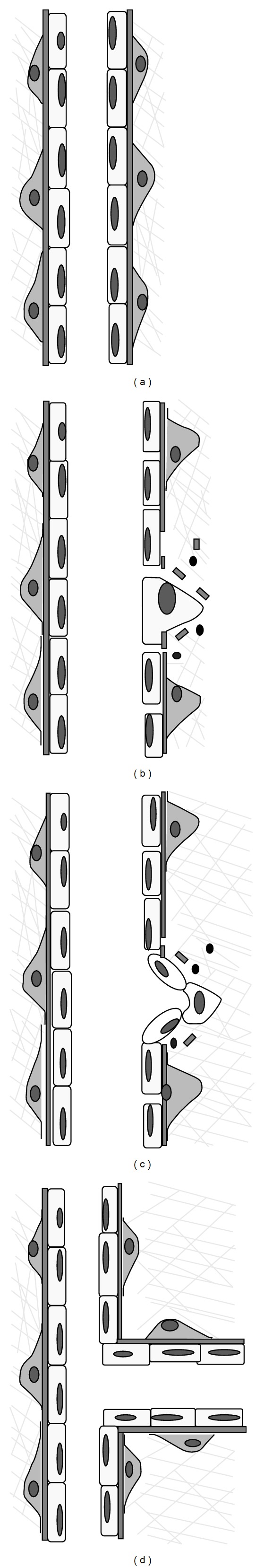
After stimulation with angiogenic factors of a quiescent vessel (a), the degradation of the basement membrane, pericyte detachment, and loosening of endothelial cell junctions occur (b). Endothelial cells begin to proliferate, migrate, and take part in formation of an immature capillary structure and deposition of a new complex basement membrane (c). Finally, pericytes are recruited thereby providing stabilization for the new vessel (d).

Angiogenesis has been studied by means of several* in vitro* and* in vivo* models, including endothelial cell cultures, chick embryo chorioallantoic membrane (CAM) assay [4], and ocular models [5]. Angiogenesis is a multistep process that generally begins when the endothelial cells switch from the “quiescent” to the “angiogenic phenotype” in response to angiogenic stimuli [6, 7] (Figure 2). Subsequently, enzymatic degradation of capillary BM occurs and vascular permeability increases leading to extravasation of blood proteins and their accumulation into interstitial collagen matrix to form a new, provisional ECM. Then, endothelial cells begin to proliferate, invade the ECM, and take part in the formation of an immature capillary structure and deposition of a new complex BM. Finally, pericytes are recruited, thereby providing stabilization for the new vessels. The soluble growth factors, membrane-bound proteins, cell-matrix and cell-cell interactions, and hemodynamic forces all act in concert to control and influence angiogenesis, and the balanced activity between specific angiogenic molecules which can initiate this process and specific inhibitory molecules which can stop it are thought to be critical for an optimal angiogenic response.

Through adhesive interactions with integrins expressed on the endothelial cells surface, the ECM orchestrates complex signalling cascades within the cells and affects many fundamental aspects of their biology, including proliferation, migration, cytoskeletal organization, cell shape, survival, and ultimately blood vessel stabilization. Moreover, matrix molecules or fragments that show pro- and antiangiogenic activity (Table 1) are critical in the onset of angiogenesis and angiogenic cytokines which directly bind matrix and require proteolytic processing to become active [8].

## 2. ECM Components Involved in Angiogenesis

### 2.1. ECM Molecules

*In vivo* studies in knockout mice for BMs genes (fibronectin, laminin, collagen IV, and perlecan) revealed significant cardiovascular dysfunctions [9–11]. In detail, several studies bear out the hypothesis that blood vessels formation and survival are connected with collagen synthesis and deposition in BM [12, 13]. Endothelial cell adhesion to ECM, via integrins-collagen I interaction, leads to activation and/or suppression of multiple signalling pathways. In human dermal microvascular endothelial cells, isolated from neonatal foreskins and anchored to collagen I, as well as in a mouse model of skin angiogenesis involving subdermal injection of Matrigel together with immortalized human cells stably transfected with VEGF165, the interaction of collagen I with *α*1*β*1, *α*2*β*1, *α*v*β*3, and *α*v*β*5 integrins on cell surfaces induces the activation of MAP kinase pathway which, in turn, supports endothelial cells survival and suppresses apoptosis [14]. In the same* in vitro* model, other investigators demonstrated that integrins *α*1*β*1 and *α*2*β*1 binding to collagen I induced suppression of cAMP-dependent PKA, following reorganization of actin fibers and changes in cell shape [15]. In mouse skin model that used VEGF-expressing cells together with packaging cells producing retroviruses encoding RhoA GTPase mutants, the endothelial cells adhesion to collagen I selectively induced activation of Src and Rho and suppression of Rac activity, which, in turn, disrupt intercellular junctions [16]. Interestingly, collagen I also contributes to coalescence of pinocytic intracellular vacuoles, which is an essential step for lumen formation [17, 18].

Type IV collagen, the main protein component of all BMs, has a crucial role in endothelial cell proliferation and cell behaviour [19]. An* in vitro* study that analysed angiogenic and nonangiogenic culture systems demonstrated the dependence of angiogenesis on secretion and subsequent extracellular deposition of collagen type IV [20].

Laminin of BM appears to be involved prevalently in the regulation of the late stages of angiogenesis: it is responsible for cessation of endothelial cells proliferation and pericytes recruitment and vessels stabilization through Notch signalling activation [21]. By using antibodies directed to laminin receptor, it has been demonstrated that laminin can activate proteinases and contribute to matrix degradation [22].

Fibronectin is a widely distributed glycoprotein and is a component of plasma in a soluble dimeric form and of cell surface and ECM in a dimeric and multimeric form and is localized in ECM underlying endothelial cells. The arginine-glycine-aspartic acid (RGD) motif was the first sequence of fibronectin found to possess cell-adhesive properties [23] and the use of the microfluidic shear devices suggested that endothelial cells adhere to fibronectin stronger than type I collagen [24]. The binding of RGD sequence to the integrins *α*5*β*1 and *α*v*β*3 in endothelial cells initiates the polymerization of fibronectin locally synthesized [25], which, in turn, regulates cell growth as demonstrated in cultures of mouse embryonic cells that lack endogenous fibronectin [26], regulates cytoskeletal organization [27], and stabilizes cell-matrix adhesion [28]. Additionally, fibronectin controls endothelial cell survival during angiogenesis* in vivo* by suppressing the activity of PKA [29]. Plasma fibronectin incorporated in the serum-free collagen gel culture of rat aorta induced a selective dose-dependent elongation of microvessels, without affecting mitotic activity of cells [30].

### 2.2. ECM Fragments: Matrikines and Matricryptic Sites

In the early stage of angiogenesis, the degradation of ECM occurs in response to angiogenic stimuli. As a consequence, matrix molecules are degraded or partially modified, soluble factors are released, and cryptic sites are exposed.

In 1960, the presence of small peptides derived from degradation of connective tissue glycoproteins was demonstrated for the first time [31]. Maquart et al. [32] introduced the term “matrikines” to designate peptides produced from degradation of ECM components, as result of enzymatic activity of proteinases produced by connective tissue cells. The same term has been then adopted to describe peptides derived by partial proteolysis of ECM surrounding microvessels. Endothelial cells produce proteinases, whose activation is responsible for degradation and liberation of matrix fragments which, in turn, can regulate cellular activity by binding specific cells receptors and by activating intracellular signalling pathways.

The term “matricryptic sites” has been coined to describe the biologically active sites that are not exposed in the mature, secreted form of ECM molecules but which become exposed after structural or conformational alterations [33].

Two classes of proteolytic enzymes appear to be mainly involved in the matrix degradation: plasminogen activator (PA)/plasmin system and matrix metalloproteinases (MMPs). The plasminogen activator/plasmin system is an enzymatic cascade involved in the control of fibrin degradation, matrix turnover, and cell invasion. The physiological activators urokinase-type PA or tissue-type PA mediate the conversion of the inactive plasma zymogen, plasminogen, to the serine protease plasmin. The latter belongs to the large serine proteinase family and can act directly or indirectly, by cleaving numerous ECM proteins, including fibronectin, laminin, thrombospondin, and von Willebrand factor [34], by activating MMPs [35], or by liberating growth factors and cytokines sequestered within the ECM [36, 37].

MMPs belong to the family of zinc endopeptidases and can exist in both membrane-bound (MT-MMPs) and soluble forms. The latter are secreted as inactive proenzymes and their activation occurs in the extracellular compartment. MMPs are produced by a variety of cells, including epithelial cells, fibroblasts, inflammatory cells, and endothelial cells, and their activity is inhibited by the family of tissue inhibitors of metalloproteinase (TIMPs) [38]. At least, five MMPs are involved in angiogenesis: MMP-1, -2, -3, -7, and -9 are upregulated in endothelial cells in a variety of physiological and pathological settings [35].

Many of matrikines and matricryptic sites, resulting from PA/plasmin system and MMPs activity, are important physiologic angiogenesis inhibitors. Among these, endostatin has been extensively studied. It is a proteolytic fragment of the C-terminal noncollagenous domain of collagen XVIII, isolated from the conditioned media of a nonmetastatic murine hemangioendothelioma cell line [39]. Its role as a local inhibitor of angiogenesis has been demonstrated* in vitro* and* in vivo* [39, 40]; nevertheless, the evidence that lack of endostatin does not affect angiogenesis in major organs suggests that this fragment is not a critical regulator of angiogenesis [41]. Endostatin acts by inhibiting endothelial cells proliferation and migration [39] and by blocking G1/S phase transition and apoptosis of cells [42, 43]. Immunoblotting analysis of endostatin treated murine brain endothelial cells revealed that endostatin induces activation of phosphatases or other regulatory signalling proteins, which may interfere with FGF effects on endothelial cells; nevertheless, this occurs only if endostatin binds to the endothelial cell surface via heparan-sulfate proteoglycans [44, 45]. Moreover, endostatin inhibits VEGF-induced endothelial cell migration in a dose-dependent manner [46].

Tumstatin, canstatin, and arresten are derived from degradation of *α*3, *α*2, and *α*1 chain, respectively, of type IV collagen. Tumstatin, generated by MMP-9 proteolysis [47], binds to endothelial cells via *α*v*β*_3_ integrin [48]. Its antiangiogenic activity is restricted to amino acids 54–132 within the 244 amino acid complete sequence [49].* In vitro* studies showed that tumstatin peptide negatively regulates endothelial cells proliferation and induces apoptosis [50] through inhibition of protein synthesis [51]. Canstatin in an antiangiogenic fragment derived from NC1 domain of the *α*2 chain of type IV collagen [52]. Recombinant canstatin selectively inhibits endothelial cells proliferation and tube formation in a dose-dependent manner [53]. Moreover, in human umbilical vein endothelial cells, canstatin inhibits the phosphorylation of Akt and focal adhesion kinase, induces Fas ligand expression, and activates caspase-dependent apoptotic pathways [54]; all these activities are mediated by interaction with *α*v*β*_3_ and *α*v*β*_5_ integrins on the surface of endothelial cells [55]. Arresten binds to *α*1*β*1 integrin and heparan sulphate proteoglycans and exerts its antiangiogenic effect in endothelial cells via inhibition of MAPK signalling [56, 57]. Arresten inhibits, in a dose-dependent manner, the proliferation of mouse retinal endothelial cells cultured on type IV collagen and stimulated with fibroblast growth factor-2 (FGF-2) [58].* In vivo*, arresten significantly inhibits neovascularization in Matrigel plug assays [59].

Tetrastatin, pentastatin, and hexastatin are derived from degradation of type IV collagen, from *α*4, *α*5, and *α*6 chain, respectively. Tetrastatin and pentastatin lack antiangiogenic activity in the CAM assay [48]; nevertheless,* in vitro* they affect angiogenesis by interacting with human endothelial cells and potently inhibiting their migration [60]. On the other hand, hexastatin administration resulted in inhibition of angiogenesis in the CAM [48] and in Matrigel plug assay [61]. Additionally, through the non-RGD-dependent *α*v*β*3 binding sites, hexastatin significantly inhibits endothelial cell proliferation [61].

Endorepellin is derived from perlecan [62] and by interacting with the *α*2*β*1 integrin receptor triggers a signalling cascade that leads to disruption of the endothelial actin cytoskeleton [63]. Endorepellin affects angiogenesis by interacting with the VEGF receptor through its laminin G-like, leading to the downstream of VEGF signalling [64, 65].

### 2.3. Angiogenic Factors

The role of VEGF as major inducer of angiogenesis is well recognized [66]. VEGF induces expression of *α*1*β*1 and *α*2*β*1 integrins in microvascular endothelial cells [67], endothelial cell migration, and proliferation [68, 69]. VEGF is not stored intracellularly but it bounds the cell surface or ECM and various MMPs [70] and PA [71] can generate diffusible, non-heparin-binding fragments. Two classes of VEGF binding sites have been identified on fibronectin: one constitutively available and the other whose availability is modulated by the conformational state of fibronectin, which, in turn, depends on heparin interaction [72, 73].

FGF-2 stimulates survival, proliferation, migration, and differentiation of endothelial cells both* in vitro* and* in vivo* and binds with high affinity to heparan sulfate proteoglycans located on the surface of most cells and within the ECM [74]. Heparan sulfate proteoglycans, particularly perlecan, modulate the binding of FGF-2 to its specific tyrosine kinase receptors [75] and protect the growth factor from proteolytic degradation by extracellular proteinases [76].

The proangiogenic activity of transforming growth factor beta 1 (TGF-*β*1) has been demonstrated in new-born mice [77] and in the CAM assay [78] and it has been confirmed in TGF-*β*1 knockout mice [79]. However,* in vitro* studies suggested that activity of TGF-*β*1 is strictly dependent on composition and organization of the ECM: TGF-*β*1 treatment elicits the formation of calcium and magnesium dependent tube-like structures, mimicking angiogenesis, in three-dimensional cultures [80], whereas it induces endothelial cells apoptosis in two-dimensional cultures [81]. TGF-*β*1 upregulates the VEGF expression and the interaction with its receptor in endothelial cells and this mechanism induces endothelial cell apoptosis and angiogenesis* in vitro* and* in vivo* [82, 83].

## 3. ECM as a Link between Angiogenesis and Inflammation

Angiogenesis and inflammation are distinct processes that can occur independently of each other, although in some cases they are codependent. Angiogenesis can stimulate and intensify the inflammatory response by providing nutrients and oxygen in inflammatory sites, and some angiogenic factors exert proinflammatory activity. Conversely, in chronic inflammation, inflammatory cells produce cytokines and growth factors that may affect endothelial cell functions.

Angiogenesis and inflammation are associated with nuclear factor kappa-B (NF-*κ*B) and angiopoietin- (Ang-) Tie2 signalling pathways. NF-*κ*B is an inducible transcription factor, whose activation regulates the expression of genes for proinflammatory cytokines, chemokines, and enzymes that generate mediators of inflammation. There is also evidence that this transcription factor is involved in the regulation of migration, proliferation and survival of endothelial cells [84], and expression of MMPs [85]. Ang-1 is secreted and incorporated into and sequestered by the ECM. By binding Tie-2 receptor expressed in endothelial cells, it does not affect the cells proliferation; however, it stimulates endothelial cells migration, sprouting, and survival and promotes recruitment of the pericytes and smooth muscle cells [86]. Mice lacking of Ang-1 and Tie-2 showed defective remodelling and maturation of the vasculature, whereas the transgenic mice overexpressing Ang-1 displayed increased vascularization suggesting a role in the formation of blood vessels during development [87]. Additionally, Ang-1 has been shown to inhibit vascular permeability and exert anti-inflammatory effects [88]. It has been demonstrated that Ang-1 can act also in nonendothelial cells, including monocytes via p38 and Erk1/2 phosphorylation and macrophages inducing their switch toward a proinflammatory phenotype [89]. Unlike Ang-1, its antagonist, Ang-2, is not incorporated into ECM and disrupts blood vessel formation in the mouse embryo [90]. Stored in endothelial Weibel-Palade bodies, Ang-2 is rapidly released in response to exogenous stimuli and promotes inflammation [91].

Angiogenesis and inflammation share ECM remodelling which, in turn, directly and indirectly influences both processes. ECM molecules and fragments, resulting from proteolysis, can act directly as inflammatory stimuli; they can influence immune cell activation and survival and proper tissue repair as well as each step of angiogenesis, as previously described.

Inflammatory cells produce a large amount of MMPs, which activate cytokines and chemokines through cleavage, regulate inflammatory cells response, and notably contribute to ECM degradation. Analysis of RNA levels expression in mononuclear cells isolated from venous blood of normal volunteers revealed that they highly express several forms of MMPs [92]. Expression of MMPs can be selectively induced by binding of bacteria to toll-like receptor 2 in monocytes and CD14, toll-like receptor 2, and toll-like receptor 1 in macrophages and is strictly dependent on the stage of cellular differentiation [93, 94].

The role of inflammation in tumor development is not less negligible: an inflammatory microenvironment can increase mutation rates and enhance the proliferation of cancer cells, contributing to tumor initiation, and innate and adaptive immune system cells can infiltrate the tumor sites, release growth and survival factors, and facilitate angiogenesis, tumor growth, invasion, and metastasis by themselves or by inducing other effector molecules. The increase of several immune cell-derived factors, such as interleukin-1 (IL-1), IL-6, IL-8, IL-10, and tumor necrosis factor alpha (TNF-*α*), has been described in tumor microenvironment: they sustain inflammatory process, contribute to tumor progression, and also exert proangiogenic activity [95, 96].

Osteopontin (OPN), a multifunctional ECM phosphoprotein-containing and RGD integrin binding domain, is limited to the bone, kidney, and epithelial linings and is secreted in body fluids including milk, blood, and urine in normal conditions, whereas it is upregulated at sites of inflammation and tissue remodelling. Chakraborty et al. [97] provided both* in vitro* and* in vivo* experimental evidences that OPN regulates Brk/NF-*κ*B/ATF-4 signalling cascades which, in turn, upregulates the VEGF expression and tumor angiogenesis through autocrine and paracrine mechanisms in breast cancer system.

Inflammatory cells release MMPs involved in the release of angiogenic factors, such as VEGF and FGF-2, and cryptic antiangiogenic factors. VEGF derived from matrix stores, as a result of MMP-9, is implicated in the angiogenic switch and tumor growth [98].

Rheumatoid arthritis (RA) is an autoimmune disease characterized by chronic inflammation of synovial joints and destruction of cartilage and bone, as well as by systemic extra-articular inflammation. The synovium that normally is relatively acellular and with scattered blood vessels, results hyperplastic, rich of inflammatory cells and high vascularized in RA. The new vessels besides provide nutrients and oxygen, also sustain the inflammation by being a source of cytokines, chemokines, and proteases [99], facilitate the ingress of inflammatory cells into the synovium, and, therefore, stimulate pannus formation. At the same time, infiltrated inflammatory cells stimulate angiogenic process, by upregulating proangiogenic factors [100, 101] and activating NF-*κ*B. Upregulation of Angs-Tie 2 [102] and VEGF [103] detected in chronic inflamed synovium and serum of RA patients emphasizes the interdependence of inflammation and angiogenesis in RA. VEGF may act as a proinflammatory mediator and as an angiogenic stimulator in RA joints: it induces the production of chemokines by endothelial cells, such as MCP-1 and IL-8 [104], which, in turn, recruit monocytes in synovial membranes. Several clinical studies demonstrated high levels of MMPs, directly involved in ECM degradation, in the systemic circulation and synovial fluid of patients with RA and their correlation with clinical activity [105, 106].

Osteoarthritis (OA) is a disease characterized by degeneration of cartilage and its underlying bone within a joint as well as bony overgrowth. Even if OA has been commonly described as “noninflammatory” disorder, in order to distinguish it from inflammatory arthritis, evidence is now accumulating that synovitis occurs and exacerbates structural damage [107, 108]. The inflammation contributes directly to angiogenesis, observed in the synovium of osteoarthritic joints [107], and these two processes contribute to pain and damage. Hypoxia, a common feature of the inflamed synovial environment, and IL-1 stimulate VEGF expression in synovial fibroblasts [109]. Also TNF-*α*, which promotes angiogenesis* in vivo* [110] and regulates the expression of MMP-9 and MMP-1 [111], is involved in OA. Thus, angiogenesis may exacerbate inflammation in OA, by facilitating inflammatory cell infiltration [111].

Psoriasis is a chronic inflammatory disease of skin and small joints characterized by excessive growth of the epidermal keratinocytes, inflammatory cell accumulation, and excessive dermal angiogenesis. Psoriasis is characterized by the overproduction of interferon gamma (INF-*γ*), TNF-*α*, and IL-17 [112]. TNF-*α* induced upregulation of IL-24 and activation of signal transducer and activator of transcription 3 (STAT3) signalling in mice keratinocytes [113]. IL-9 contributes to the development of psoriatic lesions, by inducing Th17-related inflammation and by promoting angiogenesis [114]. Keratinocytes in the psoriatic skin lesions are a source of proangiogenic cytokines, such as VEGF, whose levels have been shown to be dramatically elevated in human psoriatic skin [115] and correlate with increased levels of inflammatory cytokines and MMPs [116] and with degree of psoriasis severity [117]. Additionally, keratinocytes isolated from psoriatic skin show a strongly reduced expression of thrombospondin-1, an endogenous inhibitor of angiogenesis. Enzyme immunoassay [118] and immunohistochemistry analysis [119] revealed that keratinocytes in psoriasis express and produce MMP-1 and MMP-19, which are decreased by anti-TNF-alpha [120].

Ocular angiogenesis is an important cause for severe loss of vision in several disorders, such as age-related macular degeneration, diabetic retinopathy, retinal artery or vein occlusion, and retinopathy of prematurity. Even if the mechanism that leads to abnormal growth of new vessels in eyes remains to be elucidated, it is related to inflammation. VEGF, as well as IL-6, IL-8, and IL-10, monocyte chemoattractant protein-1 levels are higher in patients with ocular diseases, when compared with normal subjects, and significantly decreased after administration of bevacizumab, a humanized anti-VEGF monoclonal IgG1 antibody [121, 122]. VEGF intravitreal injection induced upregulation of intercellular adhesion molecule in endothelial cells and, consequently, the local adhesion of leucocytes and vascular permeability [123]. Injection of FGF into the vitreous cavity did not show the same effect of FGF mobilized by degradation of the matrix by proteolytic enzymes [123]. Conversely, macrophage depletion in mice reduced the choroidal neovascularization and was associated with decreased macrophage infiltration and VEGF protein [124].

## 4. Concluding Remarks

Overall, the literature data analyzed in this review demonstrate that, in addition to providing basic support for cells, ECM is also a critical component that allows the intercellular crosstalk and stores several important mediators which, in turn, regulate several cellular and connective components functions, including angiogenesis.

The progressive increase of knowledge concerning the cellular and extracellular mechanisms that regulate angiogenesis in normal and pathological conditions may provide the potential basis for the development of new therapeutic approaches against abnormal angiogenesis that contributes to the pathogenesis of several disorders.

## Figures and Tables

**Table 1:** ECM molecules and fragments with proangiogenic and antiangiogenic activity.

Proangiogenic	Antiangiogenic
*Intact molecules *	*Fragments *
Collagen I	
Collagen III	Arresten
Collagen IV	Canstatin
Collagen XV	Tumstatin
Collagen XVIII	Restin
Fibrillin	Endostatin
Fibulin-1	Anastellin
Fibrin/fibrinogen	Heparin binding fragments
Fibronectin	Endorepellin
Glypican-1	Endostatin
Laminin-1	Elastin derived peptides
Laminin 8	*Transient molecules *
Perlecan	Thrombospondin-1
Tenascin C	Thrombospondin-2
Tenascin X	
Vitronectin	
Decorin	
*Fragments *	
Fragment E (fibrin)	
